# “Becoming the student of the patient”: a framework for embedding cultural humility in a medical curriculum

**DOI:** 10.3389/fmed.2026.1890068

**Published:** 2026-09-04

**Authors:** Mairead Corrigan, Grainne P. Kearney

**Affiliations:** Centre for Medical Education, School of Medicine, Dentistry and Biomedical Sciences at Queen’s University, Belfast, United Kingdom

**Keywords:** cultural humility, curriculum, equity, medical education, power, social justice, cultural models

## Abstract

**Introduction:**

Cultural humility is a lifelong commitment to self-reflection of biases, redressing power imbalances in the doctor-patient relationship to develop patient-centred care and advocacy partnerships with communities, with a focus on institutional accountability. It has the potential to be transformative and address social injustices. Yet there is little published evidence on embedding cultural humility in undergraduate medical curricula and how such curricula are received. We provide a framework for how we are embedding (and continue to embed) cultural humility into a spiral curriculum from Years 1–5 with increasing complexity at Queen's University Belfast. We offer this framework for others.

**Methods:**

In developing the framework, we mapped the different dimensions of cultural humility using Tervalon and Murray-Garcia's original model of cultural humility, which allowed us to identify teaching and assessment that align to cultural humility delivered both by the authors of this paper and our colleagues. The Y1 and Y3 students were surveyed about their experiences of cultural humility teaching; the authors have also commented on anonymised student and tutor evaluative feedback relevant to the dimensions.

**Results:**

Framing cultural humility from Year 1 as their becoming the “student of the patient” resonated with students as a memorable way of being culturally humble. Students practise “becoming the student of the patient” through clinical encounters with patients and in simulated clinical experiential learning in Years 1–5. Language, both oral and written, also provides an entry point in discussions of power by developing students’ critical consciousness of how clinical language can contribute to power imbalances between doctors and patients. To date, efforts to embed cultural humility have mainly focused on the intra-interpersonal dimension, as opposed to the institutional dimension.

**Discussion:**

Cultural humility should be introduced from the start of medical training and embedded with increasing complexity and experientially throughout undergraduate education. Language is fundamental to involving students in embedding cultural humility. Our journey to embed cultural humility is ongoing; we offer an argument as to how and why power and institutional commitment needs ongoing attention.

## 1 Introduction

Medical educators have a responsibility to deliver a curriculum that is fit-for-purpose in developing students’ skills, knowledge and confidence to be able to care for patients from diverse backgrounds, for example race, ethnicity, gender, disability, religion, sexual orientation and socioeconomic status. Global health inequities experienced by groups from minoritised and vulnerable backgrounds, which were exposed and deepened by the COVID-19 pandemic (1), makes ability to care for diverse patient populations a matter of social justice and equity.

Various models have been developed to aid healthcare practitioners care for diverse populations and address health disparities. One of the most long established and arguably the most well-known is cultural competency. This was developed in 1989 by a Native American social worker Cross (2) along with colleagues, to improve delivery of culturally relevant services to minority children of colour and their families. Cultural competency requires development of behaviours congruent with the culture of the person, including learning about different cultures in a way that is inclusive of an individual's racial, ethnic, religious, or social group. It requires attitudes that are unbiased and less ethnocentric and flexible policies to enable professionals to function effectively cross-culturally. It is a developmental process that takes time and requires engagement by everyone in an organisation which should have minority staff in its recruitment. Yet, its application in practice has been at the level of the individual practitioner and their ability to interact effectively with clients, with less attention at the institutional level. Consequently, much of the focus has been on practitioners enhancing their cultural knowledge, leading to criticisms of the model for stereotyping and othering minority racial and ethnic cultures and focusing on cultural difference (3).

These criticisms of cultural competency led to the development of cultural safety as an alternative. Developed by Dr. Irihapeti Ramsden and Māori nurse colleagues in Aotearoa in the 1990s to tackle the health inequities of Indigenous Māori, it was legislated into New Zealand nursing and midwifery education in 1992. Cultural safety is defined as “a focus for the delivery of quality care through changes in thinking about power relationships and patients’ rights” (4, p493). Unlike cultural competency, the focus of cultural safety is on ‘power’ imbalances between practitioners and patients, which can be because of differences in status, cultures, colonial histories, ethnicities or levels of material advantage. Practitioners are encouraged to reflect on how these might contribute to stereotypes and assumptions that are personal to them, as well as those which are embedded institutionally in the healthcare profession and in wider society. It requires them to develop a critical consciousness of societal injustices and formulate these reflections into an action plan to tackle health inequities (5). Cultural safety tends to be embedded in training for health professionals and service level providers in nations with indigenous populations (6). Patients have the right to determine if they feel safe (physically, mentally, socially, spiritually and culturally) with the healthcare that is provided, not the healthcare practitioner (4). Developing a healthcare environment where patients feel culturally safe can be challenging because of the focus on structural change (5).

Criticisms of cultural competency also led to the development in 1998 of an alternative model, cultural humility, by two African American clinicians, Tervalon and Murray-Garcia who were working on racial health disparities and physician training in the United States. Cultural humility is defined as, “a lifelong commitment to self-evaluation and self-critique, to redressing the power imbalances in the patient-physician dynamic, and to developing mutually beneficial and non-paternalistic clinical and advocacy partnerships with communities on behalf of individuals and defined populations” (7, p117). It shares similarities with cultural safety by requiring physicians to reflect on and be aware of biases that are personal and embedded in the provision of healthcare, which can lead to inequitable treatment of minority populations. Physicians are encouraged to adopt self-reflection as a lifelong “way of being” (7, p123). Like cultural safety, it addresses power imbalances by encouraging physicians to relinquish the role of expert, to admit when they don't know and instead to become the “student of the patient” (7, p121), by learning for example about their patients’ culture and health beliefs and working collaboratively with them to incorporate these into how they care for them. Cultural humility is not a sign of weakness or lack of knowledge but is an ethical expression of expertise that humanises doctors (8). The purpose is to provide optimal care that is respectful and culturally sensitive based on partnership and mutual empowerment (9). These characteristics of cultural humility align with education and training that is person-centred and tailored to the individual patient. In the UK, the Medical Schools Council (MSC) Equity, Diversity and Inclusion (EDI) Alliance, which provides guidance to medical schools on embedding EDI into curricula, encourages including cultural humility in curricula to reduce stereotyping (10). For this reason and given that cultural humility is practitioner facing and internally oriented, this aligns well with undergraduate medical education (8).

### 1.1 Embedding cultural humility in curricula

To be sustainable, cultural humility needs to be embedded longitudinally in curricula and “threaded” throughout (11). This can be challenging because of limited timetable space, especially for subjects like cultural humility that are perceived as a “soft” skill (12).

There exist strategies and toolkits of how to embed cultural humility longitudinally at an undergraduate level for example in nursing (13), veterinary sciences (14), psychiatry (15), social work practice (16), pharmacy (17) and at a postgraduate level for hospital doctors (18) and for family medicine (19). In undergraduate medicine, cultural humility tends to be delivered in “silos” through standalone teaching sessions (19–22). There is an absence of guidelines or practical examples of how to embed cultural humility longitudinally in undergraduate medical education curricula (23, 24).

At Queen's University, Belfast, we have been embedding cultural humility in our medical curriculum, with increasing complexity across Years 1–5, as part of a spiral curriculum which was introduced in 2020 (25). In this article, we describe an approach to cultural humility that includes teaching developed by the authors and their academic colleagues which align with cultural humility. We will report how this approach to cultural humility has been received by medical students and those teaching through a cultural humility lens. This article presents our learning for others interested in considering how they could begin their journey in embedding cultural humility in medical curricula.

### 1.2 Pedagogical framework: cultural humility

Cultural humility has become increasingly popular in healthcare education and training, with its inclusion in contemporary definitions of cultural competency, which has been refined as a lifelong process model of self-awareness and critical awareness of power structures while working in partnership with patients rather than claiming an expertise over others (26). Cultural humility is also regarded as important for cultural safety and for quality intercultural relations because it involves “a process of self-reflection to understand personal and systemic conditioned biases, and to develop and maintain respectful processes and relationships based on mutual trust…[it] involves humbly acknowledging oneself as a life-long learner when it comes to understanding another's experience” (27).

Among academic publications, Tervalon and Murray-Garcia's article remains the most frequently cited source for the model of cultural humility (16). Their model is multilevel, comprising of lifelong self-reflection, patient-centredness, community-based care and advocacy, and institutional consistency. Yet, little attention is given in the literature to the dimensions of advocacy and institutional consistency in cultural humility, with training in cultural humility focusing more on the intrapersonal and interpersonal dimensions of the model—self-reflection and patient-centredness. A recent concept analysis of contemporary understandings of cultural humility confirmed this when it identified its attributes to be a lifelong process of learning that involves self-reflection and critique, being egoless and open to others, affirmation of cultural differences, immersion into an individual patient's point of view, supportive interactions and flexibility based on partnership, mutual empowerment and respect. Its antecedents were diversity and power imbalance while the consequences were mutual empowerment, partnerships, respect, optimal care, and lifelong learning (28).

Advocating for marginalised patients requires students to develop a critical consciousness of social injustices not only of healthcare inequities that are behavioural and biological, but that are structurally embedded in society and healthcare (9, 23, 29, 30). Cultural humility also needs to be extended at an institutional level, by questioning whose knowledge is dominant and by challenging hierarchy to foster true collaboration through “co-creation, co-knowledge and co-accountability”, that “showcases the value of collaboration and ensures open-mindedness, attunement to uncertainty and responsiveness to complexity” (8, p606). Cultural humility has also been criticised for lacking empirical evidence about its impact for patients (23). Its inclusion in contemporary understandings of cultural competence have led to criticisms of cultural humility for being no different from cultural competence other than offering “semantic appeal” (3, 23). There also remains a pedagogical divide between those who reject cultural competence because of the risk of stereotyping (3, 9, 16, 30) and those who claim that both humility and cultural knowledge are required and that one needs to learn about other cultures to be able to function cross-culturally, whilst acknowledging culture as individual and dynamic (31).

Whilst we acknowledge the limitations of cultural humility, described above, it remains core to developing a quality intercultural presence with a focus on social justice. In this article, we describe our work embedding cultural humility through an undergraduate medical curriculum. We map our curriculum against Tervalon and Murray-Garcia's original four-dimensional model: 1) self-reflection and lifelong commitment to learning; 2) patient-focused interviewing and care; 3) advocacy and community care; 4) institutional consistency, demonstrating how it can be embedded as a multilevel construct.

### 1.3 Learning environment

The development of a new spiral curriculum beginning in 2020 at Queen's University Belfast, known as C25 (25), afforded an opportunity to introduce and embed cultural humility. Much of the literature discussed above focused on applications in postgraduate medicine or other disciplines. Our intention was to bridge from theory to application in practice for an undergraduate medical curriculum.

Positioning cultural humility into a spiral curriculum was important to us. A spiral curriculum integrates subjects horizontally and vertically and revisits them with increasing complexity, which can facilitate embedding diversity into a curriculum (32). Several of the components of the C25 curriculum created the conditions to embed cultural humility. Case-based learning, with links to real life clinical scenarios, provided an anchor to introduce teaching on aspects such as racism and sexism, which were discussed further within facilitated tutorials. The aim to increase time spent in Primary Care (Family Medicine) up to 25%, not only created the setting for the introduction of cultural humility but provided a scaffold of experience-based learning, that aligned with ongoing development of cultural humility. The introduction of helical themes, such as “Global and Population Health” or “Achieving Good Medical Practice”, helped embed content on diversity and professionalism throughout the curriculum. Whilst in the early planning stages, cultural learning was planned to sit within a cultural competence model (25), developments in the literature and our learning increased our focus on cultural humility on implementation.

Several overarching principles informed our developments. Co-creation with students and where possible with community organisations was present throughout. Students and/or community organisations were therefore involved in co-design and co-delivery of the sessions, and where feasible, in re-design based on gathered evaluative data and feedback. There was a focus on learning through experience, ideally with patients in practice or where this wasn't possible or feasible, with experiential learning in simulated learning environments. There is ongoing work in diversifying the curriculum, working towards a goal of decolonising the learning environment, including how the curriculum is delivered, assessed and experienced. Finally, there was a focus on pedagogy and research both to inform what we do but also in creating an evidence-based and experience-based framework that may be usable for others.

Our learning objective was to embed cultural humility into our curriculum, progressively and in a spiral way. Recipients of this learning, as described below, include all students from Years 1–5 but importantly also include faculty within the medical school and those who teach students on clinical placements.

## 2 Methods

Ethical approval (MHLS 21_98- Amendment 1) was received to collect data from Year 1 and Year 3 medical students at QUB of their reflections on cultural humility teaching developed by the authors during the first iterations of the teaching in the 2021–23 academic years. Both year groups were invited to complete an online anonymous survey to assess current knowledge, attitudes and perceived applicability of cultural humility, with a specific focus on power in Year 3. The Year 1 students were also asked for their consent for the authors to access their written blogs on cultural humility which formed part of their assessment and which were anonymized for the study.

We gathered survey data from 188 Year 1 students, out of a possible 312, with 10 students from this cohort giving us permission to analyse their personal blogs (we have not quoted from these directly in this paper as they related more to experience of early clinical contact rather than cultural humility specifically). A small number engaged ‘live’ with the optional Year 3 teaching; four completed the survey associated with this teaching.

The authors also analysed anonymised written evaluations of teaching on cultural humility developed by them, from students and tutors, as part of the quality assurance process for which no ethical approval was sought. The authors provide a summary in their own words of relevant feedback, with no direct quotes taken from students or tutors.

Given that self-reflection is essential for cultural humility, it is important that we reflect on our positionality. The authors are longstanding medical educators. MC is a sociologist, who as academic lead for diversity, equality and inclusion in medical education at QUB has been leading on diversifying the C25 spiral curriculum. GPK is a Clinical Professor and Academic General Practitioner with an interest in social justice in medical education.

## 3 Results—embedding cultural humility into an undergraduate medical curriculum

We used Tervalon and Murray-Garcia's original four dimensions as a framework to embed cultural humility into a spiral curriculum. Taking each dimension in turn, we will describe what they entail and what our application looked like in the curriculum with evaluative reflections and commentary. In addition to the initiatives we developed in the curriculum, we also include learning practices which predated the C25 curriculum which align with a pedagogical cultural humility framework. We recognise that much learning traverses the dimensions. For the purposes of this article, we have positioned these where we felt they fit best. We will continue to refer to Tervalon and Murray-Garcia's original work on cultural humility as a model and our approach to apply it as a framework. Our guiding ethos is becoming “the student of the patient” (7, p121), both as an aim and the means to achieve this aim; sometimes this is explicit and other times more implicit.

### 3.1 Self-reflection and the lifelong learner model

This first dimension of cultural humility—self-reflection and lifelong learning, is perhaps the best known. Tervalon and Murray-Garcia suggest a starting point of recognising and respecting the culture of others by consciously thinking about our own culture, suggesting also the need for those leading the learning to model this. They foreground the need for learners to be “flexible and humble enough to let go of the false sense of security that stereotyping brings … to assess anew the cultural dimensions of the experiences of each patient … to say that they do not know when they truly do not know” (7, p122). In this dimension, we explicitly use the terminology of becoming “the student of the patient” (7, p121).

In considering lifelong learning, we recognise a need to continuously develop approaches to cultural humility through research and evaluation, and through sharing our reflections on this (see Table 1).

**Table 1:** Embedding self-reflection and lifelong learning developments into medical curricula.

Year	Applications	Student/Educator Reflections	Author Commentary
Self-reflection			
1	Workshop ‘Have You Ever,’ inviting students to consider (in a safe space) their own cultural background including race, gender, and patienthood and using the “5 Rs model” (18) as a framework to self-reflect on unconscious biases and cultural humility in their summative assessment personal blogs. Bespoke video explaining cultural humility and how to link it with early clinical contact (all co-created with a Year 3 student).	“It reiterated for me the importance of reminding ourselves of not just our similarities, but our differences and how aspects of people's personal lives are not always obvious from the outer surface.” Year 1 student (survey data) “I feel like cultural humility is a long overdue model to be adopted in medical schools especially in the increasingly multicultural society we now live in. Several issues can arise in medical practice as a result of unconscious bias and stereotyping by medical practitioners, culture humility should aid in alleviating these issues.” Year 1 student (survey data) When asked, ‘How important do you think cultural humility is in your clinical practice?’ Year 1 students rated it 4.83/5 and one commented in survey, “It's not something you can essentially just teach. It is something you need to experience and work on throughout your career. People need to be open-minded and that takes time to change alongside a lot of other beliefs we have grown up with.”	This student feedback suggests understanding of the need for models such as cultural humility, and the importance of self-reflection within it.
1	Early clinical contact when students who are recruited from Family Medicine visit patients with chronic health conditions in their home. This provides an opportunity for students to practice becoming ‘the student of the patient’ (7).		This offers an opportunity for students to practice patient-centred interviewing and cultural humility.
1	Clinical Communication workshop on cultural competence.		Students reflect on their culture and multiple identities, unconscious biases and stereotyping for doctor-patient interactions, clinical communication and patient outcomes.
Lifelong learning			
Throughout all Years	Actively using the term cultural humility with students and staff, helping them connect their learning on cultural humility in the curriculum and consider it as ongoing learning (rather than point in time).	“… some of the phrasing—such as—thinking of the patient as your teacher was a great way of explaining it.” Year 1 student.	This comment underlines a grasp of the ethos that we have built much of the framework on.
Throughout all Years	Evaluation and research into approaches, using this learning for further development. Sharing ideas through publications and presentations.		Our integration of cultural humility in the Year 1 workshop and early clinical contact/Family Medicine scheme, described above in this table, was cited as good practice in the Medical Schools Council “Decolonising medical and dental curricula” (10), on how to embed cultural humility in an existing module.
Learning practices which predated the C25 curriculum, and which align with a pedagogical cultural humility framework			
−Reflective Practice Portfolio, inviting students to reflect on bias and diversity through Years 1–5			

### 3.2 Person-focused interviewing and care

In this second dimension, Tervalon and Murray-Garcia suggest use of patient-focused interviewing, to encourage the patient in “telling of his or her own illness or wellness story”. This helps overcome what they consider to be any unrealistic need to understand the culture of every group, as the patient is in control of how much culture does or does not play into the particulars of what is being discussed and/or the role of intersectionality. The learner or health care professional rather than being the expert in this interviewing, instead is explicitly guided by the ethos of “becoming the student of the patient” (7, p121), demonstrating their humility and enabling partnership (Table 2). We developed Tervalon and Murray-Garcia's language from patient-focused to person-focused, to include their social and cultural context.

**Table 2:** Embedding person-focused interviewing and care developments into medical curricula.

Year	Applications	Student/Educator Reflections	Author Commentary
3	“Power in clinical communication” session looking at power in both oral and written communication ‘norms’ in medicine (33).	“I think being conscious of the language we use is incredibly important, as our descriptions will always impact our perceptions of patients. Encouraging staff to reflect on their language choices allows us to challenge the connotations or perceptions associated with turns of phrase that are common both in medicine and in everyday conversation.” Year 3 student.	This student comment suggests their understanding of the need to question everyday clinical language due to its potential impact on relationships with patients.
1	Developed pedagogical framework reflecting on the centrality of what is traditionally taught as ’social history’ (34).		This framework has informed communication skills teaching in other medical schools (*personal communication*).
1–4	Case-based learning, which links to real life clinical scenarios.		The cases have been co-created with students and some with community organisations and continue to be updated in partnership with students. The cases also provide a framework for tutorials on, for example, ‘Gender, Race and Ethnicity’ and ‘The Social Determinants of Health’. In their module evaluations, students frequently report Case-based learning as an example of where they experience diversity in the curriculum.
1–2	Learning from both patients and practitioners with similar ‘lived experience’ to those featured in the cases, through overview lectures for Case-based learning.	In evaluative data, a Year 2 student commented how they found it useful when teaching focused on the more ’social’ aspects as sometimes the clinical focus removed any emotion.	This comment by a student indicates their understanding of the need for a holistic approach in learning about people.
Learning practices which predated the C25 curriculum, and which align with a pedagogical cultural humility framework			
−Students work with simulated participants in developing person-centred clinical communication skills through role-plays and in their Objective Structured Clinical Examinations (OSCEs) assessments.			

### 3.3 Community-based care and advocacy

Tervalon and Murray-Garcia bring focus to learning community-based care and within this, the role of advocacy skills. They quote Evans (7, p121) who suggests that doctors in training need awareness beyond the individual, towards the community they live in, including policies and practices that impact the social determinants of health. For them, this includes and moves beyond what community-based doctors do, into what community-based organisations do; revisiting self-reflection and humility through recognition of the expertise and advocacy work happening in communities (Table 3). In this dimension, the ethos of becoming “the student of the patient” (7, p121) is more implicit.

**Table 3:** Embedding community-based care and advocacy developments into medical curricula .

Year	Applications	Student/Educator Reflections	Author Commentary
1–5	Increased time in Family Medicine clinical placements.	Year1 student when referring to her Family Medicine tutor in evaluative feedback described how watching the tutor's approach has influenced how the sort of doctor she will be in the future, specifically in how she shows patients that she has time for them	
2, 4	Community activists sharing lived experience within the curriculum e.g., domestic abuse teaching and co-creating Case-based learning.	In evaluative data, a Year 4 student commented on how important it was to look at the underlying causes of domestic abuse and in considering what language is used in consultations where domestic abuse is discussed.	This student demonstrates their learning on the role of language in clinical communication.
3	“What is the hidden curriculum?” teaching session for students as they start clinical placements, under the “Achieving Good Medical Practice” helical theme (See Learning Environment).		In their Reflective Practice Portfolios, students frequently reflect on experiences which fall under the hidden curriculum; knowledge of the hidden curriculum is useful to contextualise their experiences.
2	Voluntary workshop for minority ethnic students on challenging racism on clinical placement co-created and led by students.		Students discuss racism and how to advocate for themselves.
Learning practices which predated the C25 curriculum, and which align with a pedagogical cultural humility framework			
−Curriculum in partnership with community-based organisations e.g., deaf awareness sessions, with the WAVE Trauma Centre who offer care and support to those bereaved, injured or traumatised through the ‘Troubles’, the thirty-year conflict in Northern Ireland. −Student selected components, often partnering with community-based organisations. −Student involvement on EDI committees and other committees in activist roles.			

### 3.4 Institutional consistency

The architects of cultural humility advocate that the same skills to be developed at the level of the individual learner also need to be represented at the institutional level by those who are developing the learners (7). They suggest that the need for “self-reflection and self-critique” as reflected in the first dimension of cultural humility must be visible in the institution. In this dimension, the ethos of becoming “the student of the patient” (7, p121) is more implicit but still used to guide the implementation. This dimension is not mentioned in the literature as much as the others (see Table 4).

**Table 4:** Institutional consistency in medical curricula.

Applications	Author commentary
Embedding Challenging Exclusions as an agenda item in all educational committee meetings to promote discussion of how processes, policies and structures for example may exclude minoritised students	This is a recommendation in the MSC EDI Alliance document ‘Active inclusion: Challenging exclusions in medical education’ (35)
Embedding diversity in student module evaluations to capture student experiences of diversity in the curriculum	This aims to capture recommendations from students, especially minoritized students using their lived experiences, to improve diversity in the curriculum.
Embedding diversity in staff peer review pro formas	Educators self-reflect on how they can enhance diversity in their teaching
Clinical educators were trained in cultural humility to enable them to embed it in early clinical contact. This training took place during the annual Family Medicine tutor's training session.	An experienced Family Doctor discussed their introduction to ‘power’, even sharing an example of where they now recognised it in their everyday clinical practice.
Training of staff both in the medical schools and in clinical placements through the lens of cultural humility (academic, clinical, and professional support staff) e.g., a cultural humility lens was used to design staff training on the Medical Schools Council Faith Guidance (36) including co-design and co-delivery with students.	Following this training, in discussion with one of the leads for clinical placement teaching, an infographic summarising relevant points on the Faith guidance was produced which has been added to student inductions and sent to staff involved in teaching.
Diversifying curriculum e.g., inclusion of different skin tones in Year 3 dermatology teaching and Case-based learning co-created with students.	Faculty-led staff training on diversifying the curriculum; pro-forma for staff peer review of teaching encourages reflections about diversity in their teaching; student evaluations of teaching contain questions about their experience of diversity in the curriculum.
Ongoing work in decolonising curriculum e.g., diversifying Simulated Participant recruitment, greater visibility of previously under-represented groups in teaching and research (37); history of racism in medicine.	Medical school commitment to decolonising curricula, by reframing structures around teaching e.g., training students to care across gender, race etc. and increasing visibility of under-represented groups e.g., actively involving female Simulated Participants.
Active Bystander Training (students & staff) - Introduction to the framework in Year 1. Revisit using experiential learning in “Forum Theatre” in Year 5 (38).	This provides an opportunity to reflect on unconscious biases and how to challenge and/advocate if experiencing/witnessing for example racism and sexism on campus and on clinical placement.
Online open-access EDI training, which is mandatory for all Family Medicine tutors (39).	Co-created between the two medical schools, and the postgraduate deanery in Northern Ireland, working alongside a medical student. This is deliberately freely available and open access for any interested parties globally (can be accessed through reference 39).
EDI training for clinicians, academics and Simulated Participants involved in medical student assessments.	Material addresses unconscious bias and stereotyping.
Half-day workshop on decolonising the curriculum attended by staff and students from Northern Ireland's two medical schools.	Aims of the session were to share good practice of decolonising the curriculum and to develop collaborations.
Learning practices which predated the C25 curriculum, and which align with a pedagogical cultural humility framework - Reflective Practice Portfolio as a continuum of self-reflection. - Interprofessional Schwartz rounds. - Involvement of real patients in Finals OSCEs (40). - Funded summer studentships for students to work alongside staff on EDI topics in relation to both education and research.	

## 4 Discussion

By adopting Tervalon and Murray-Garcia's model to provide examples of teaching that aligns to cultural humility delivered both by the authors of this paper and our colleagues, we demonstrate a framework of how cultural humility can be embedded creatively and recurrently with increasing complexity throughout a spiral curriculum. Students are introduced to cultural humility from Year 1, by fostering awareness of their positionality, privileges and unconscious biases through self-reflection and purposeful discussions of cultural humility. They are introduced to the construct of power through discussions for example on intersectionality, the history of medicine and social determinants of health. We explicitly use the term ‘cultural humility’ from the start of their studies, as part of their growing ‘lexicon’ of new language so that they consider its importance alongside their other new clinical terminology, and to sensitise them to identify it when enacted by their educators. Framing cultural humility as an opportunity to become the “student of the patient” (7, p121) resonated with students as a memorable and practical way of interacting with patients in a culturally humble way. Alongside learning the concept and language of cultural humility, they can practise “becoming the student of the patient” through early clinical encounters with patients in their home, through Hospital and Family Medicine clinical placements and in simulated clinical experiential learning as they progress from Years 1–5.

The concept of power is particularly challenging and complex, requiring careful consideration. Discussions of power increase in complexity from Year 3 onwards, as students’ clinical contact with patients increase. Discussions of the power of language in the form of “labels, framing, descriptions and even metaphors” (12, p557) can be a useful starting point. We chose to examine the power of clinical language that potentially can disempower patients and cause unintentional harm, through an online optional teaching session with students (33). Online delivery can provide a safe space to discuss complex topics (12). We adopted a creative approach to highlight the power of clinical language by scripting a role play and mock patient notes, which staff facilitators enact to prompt student discussions. Though numbers were low, students who engaged with the online session valued it (33) (Table 2). Embedding discussions of power imbalances in the curriculum remains work in progress.

Training on being an active bystander in challenging for example racism, develops students’ advocacy skills. This is embedded progressively with increasing complexity from classroom discussions (Year 1), to empowering Black Asian and Minority Ethnic students to challenge racism on clinical placement (Year 2), culminating in experiential simulated active bystander training to prepare students to challenge racism in the workplace led by academic colleagues (Year 5) (38). Institutional consistency is promoted by adding a fixed agenda item to medical education committee meetings promoting active inclusion and challenging exclusions, based on recommendations by the MSC EDI Alliance (35). This provides an opportunity for staff and senior leaders to discuss for example how processes linked to teaching, assessment and student support may potentially exclude or discriminate against some students from minority backgrounds and to develop actions to actively include. Diversity is further embedded in the quality assurance processes of student evaluations and peer review of teaching, to improve diversity teaching in the curriculum that reflects the lived experiences of students, especially those from minoritised backgrounds (Table 4).

Diversity training for staff provided an unintended opportunity for some Family Medicine doctors to reflect on power imbalances in their own interactions with patients. Family Medicine professionals on the front lines may be uniquely positioned to be leaders in developing and evaluating approaches to providing effective culturally humble care (19).

### 4.1 What this adds for medical education

Our experience adds several higher-level points of value both to the medical education literature and for educators keen to develop this model and apply this framework in their context. We hope to add to the early evidence base for using cultural humility as a framework in undergraduate medical education so that its uses/limitations can be further understood and applied. We found that language is fundamental to involving students in the journey of embedding cultural humility. Framing cultural humility as their becoming ‘the student of the patient’ was a role that students identified with that helped them to understand the importance of learning from patients. Language, both oral and written, also provides an entry point in discussions of power by developing students’ critical consciousness of how clinical language can contribute to power imbalances.

With any framework, there is a risk of presenting abstract offerings, in a ‘tick-box’ manner. Cultural humility learning needs to be embedded and threaded (11) into curricula as we have presented in the above Tables. The Year 1 cultural humility workshop (Table 1) is included in the MSC EDI Alliance guidance document on decolonising medical and dental curricula (10), of how cultural humility can be embedded into an existing module that could be adopted by other institutions. Other learning development strategies that we have adopted from the literature include a co-creation approach with students and with community representatives that aligns with good practice (41), as well as longitudinal experiential learning, teaching on the history of medicine and including a mix of mandatory and optional teaching (12).

Mapping the curriculum against Tervalon and Murray-Garcia's model allowed us to identify teaching developed by colleagues which aligns with cultural humility, and how it can be integrated into existing teaching sessions, which alleviates the potential strain on time and resources of developing material from scratch (42). This mapping also aids identification of gaps for future development where needed, which we discuss further below. Whilst we have mapped cultural humility to the different dimensions of Tervalon and Murray-Garcia's original model (7), this is over simplistic in practice and there is much overlap and necessary revisiting of the dimensions. We have deliberately included several overarching concepts such as the principle of co-creation and available funding for students to work on projects (Table 4), as well as pragmatic details to allow educators to consider how this might be transferable to their settings.

### 4.2 Areas for further development of the cultural humility framework

Whilst we have found our developments around cultural humility in our medical curriculum to be well received and rewarding, current cultural context necessitates further and bolder development to realise its potential to be transformative. Foronda argues for particular attention to power imbalances, developing critical consciousness and advocacy for social injustices (13). Ezell describes how global challenges to Diversity, Equity, Inclusion and Belonging work and allegations of clinical “wokefication”, can contribute to a “chilling of academic freedom” and to persistent racial inequities and social injustices (24). It is against this backdrop that Ezell calls for a reframing of cultural humility, as core to doctors’ training rather than being viewed as ancillary. Embedding cultural humility in curricula could mirror how the “habit of professionalism” (29, p787) is transmitted to students, developing a professional identity that is embodied and lifelong. Humility needs to be embedded in professional conversations and practices with physicians and students recognising limitations to an individual's knowledge, being “confident” about embracing humility (43) and receiving training in how to be “respectfully curious” (44).

A very important area for ongoing consideration is the lack of evidence about how to assess cultural humility in the curriculum. In the MSC EDI Alliance's decolonising document (10), our work on how cultural humility could be assessed was cited as good practice, whereby we ask students to use a cultural humility lens in their early clinical contact assessment blogs, as well as incorporating it into Reflective Practice Portfolio in Years 1–5 (Table 1). Whilst this lens may work well in reflective elements, increased focus on Single Best Answer type questions, as used in the National Licensing Examination in the UK (45) would be more challenging. For OSCEs, working alongside simulated participants could be a mode to help educators assess humility-based principles (40).

Against this backdrop, we consider our development of cultural humility in our medical curriculum as ongoing. By way of next steps, current toolkits and strategies for embedding cultural humility tend to ignore power and institutional accountability (16). One way in which we have been promoting institutional consistency is by linking diversity in the curriculum with quality assurance processes. This could be expanded by including questions that draw attention to power in faculty meetings for example, “How do we *actively* address inequalities both internally (i.e., policies and procedures) and externally (i.e., legislative advocacy)?” (30, p176). As advocates for cultural humility, we acknowledge the limitations of our knowledge and experiences as white, middle-class academics, by actively co-creating teaching and learning resources with students and community groups. We are continuing our work towards decolonising the curriculum (10). We recognise the need, however, for cultural humility to be extended at an institutional level, by questioning whose knowledge is dominant. As the least developed of all the dimensions, this begs the question to what extent can institutional consistency be realistically delivered in a curriculum?

There are other challenges with institutional consistency. While cultural humility, cultural competency and cultural safety were originally developed to address the effects of colonialism, racism, and systemic inequities on specific communities, they have since been adapted to include a broad understanding of culture that includes for example religion, gender identity, sexual identity, socioeconomic status, and more, which when combined with intersectionality, would render every therapeutic relationship cross-cultural (16). However, applying these models beyond race requires careful adaptation, as the nature of marginalisation differs between groups and cannot be understood through a single lens. Moreover, embedding them within an institution can also reduce attention to the structural conditions and power relations that gave rise to these models in the first place. This risks moving from institutional accountability and addressing inequity towards focusing on the interpersonal behaviours and attitudes of individual practitioners. Consequently, these models risk being transformed from a focus on confronting systemic injustice and health inequities to focusing on intra-and interpersonal behaviours and practices. We recognise the need towards a more strategic focus institutionally, more aligned with addressing inequities at a structural level.

### 4.3 Limitations

In evaluating our efforts to embed cultural humility to date, we have relied on self-reports from students and staff, that may reflect people who have a higher level of engagement with cultural humility. Evaluations of standalone cultural humility teaching sessions have been shown to demonstrate improvements in medical students’ self-awareness of biases and their psychological flexibility in being able to connect with patients as unique individuals (20, 21, 46). Whilst we include examples of good practice, we do not claim to offer best practice. Embedding cultural humility into a medical curriculum is based on an assumption in the literature that it brings benefit for patients. There is a lack of empirical data about the impact of cultural humility for patients and what constitutes best practice in embedding cultural humility (23). Future work on cultural humility is needed to understand how to meaningfully measure the impact of cultural humility teaching and training, for patients’ healthcare experiences, health outcomes and health disparities (16, 21, 42, 47). Additionally, our claim of spiralling increasing complexity as the student moves up through the clinical years is stated from our perspective as educators. An alternative view may be that the first dimension, with a focus on an individual student may be highly complex where that student has faced exclusion or discrimination. We accept that nuance and experience may be lost in our framework through our efforts to simplify it to promote its transferability to other institutions.

## 5 Conclusion

We have described what embedding cultural humility, creatively and immersively, through different modules from Years 1–5 of a medical curriculum can look like. Our approach includes a combination of specific teaching on cultural humility, embedding it in existing modules and identifying teaching delivered by colleagues that aligns with cultural humility principles. Introducing it early and deliberately into medical curricula is something tangible we can do as medical educators to enable medical students to develop a professional identity that is culturally humble and foster activism for social justice. In our approach, cultural humility is reinforced and develops in complexity as students progress in their studies, as they increase their clinical contacts with patients. The ethos of becoming “the student of the patient” (7, p121) is used throughout as both the aim and the means to achieve the aim.

Just like the concept of cultural humility itself, embedding it is a lifelong and reflective practice. Our journey embedding cultural humility is continuing.

## Data Availability

The raw data supporting the conclusions of this article will be made available by the authors, without undue reservation.
