# Correction: “Becoming the student of the patient”: a framework for embedding cultural humility in a medical curriculum

**DOI:** 10.3389/fmed.2026.2002952

**Published:** 2026-09-30

**Authors:** Frontiers Production Office

**Affiliations:** Frontiers Media SA, Lausanne, Switzerland

**Keywords:** cultural humility, curriculum, equity, medical education, power, social justice, cultural models

There was a mistake in Table 4 as published. The column ¨Student/Educator Reflections¨ was included by mistake and has been removed. The corrected Table 4 appears below.

**Table 4:** Institutional consistency in medical curricula.

Applications	Author commentary
Embedding Challenging Exclusions as an agenda item in all educational committee meetings to promote discussion of how processes, policies and structures for example may exclude minoritised students	This is a recommendation in the MSC EDI Alliance document ‘Active inclusion: Challenging exclusions in medical education’ (35)
Embedding diversity in student module evaluations to capture student experiences of diversity in the curriculum	This aims to capture recommendations from students, especially minoritized students using their lived experiences, to improve diversity in the curriculum.
Embedding diversity in staff peer review pro formas	Educators self-reflect on how they can enhance diversity in their teaching
Clinical educators were trained in cultural humility to enable them to embed it in early clinical contact. This training took place during the annual Family Medicine tutor's training session.	An experienced Family Doctor discussed their introduction to ‘power’, even sharing an example of where they now recognised it in their everyday clinical practice.
Training of staff both in the medical schools and in clinical placements through the lens of cultural humility (academic, clinical, and professional support staff) e.g., a cultural humility lens was used to design staff training on the Medical Schools Council Faith Guidance (36) including co-design and co-delivery with students.	Following this training, in discussion with one of the leads for clinical placement teaching, an infographic summarising relevant points on the Faith guidance was produced which has been added to student inductions and sent to staff involved in teaching.
Diversifying curriculum e.g., inclusion of different skin tones in Year 3 dermatology teaching and Case-based learning co-created with students.	Faculty-led staff training on diversifying the curriculum; pro-forma for staff peer review of teaching encourages reflections about diversity in their teaching; student evaluations of teaching contain questions about their experience of diversity in the curriculum.
Ongoing work in decolonising curriculum e.g., diversifying Simulated Participant recruitment, greater visibility of previously under-represented groups in teaching and research (37); history of racism in medicine.	Medical school commitment to decolonising curricula, by reframing structures around teaching e.g., training students to care across gender, race etc. and increasing visibility of under-represented groups e.g., actively involving female Simulated Participants.
Active Bystander Training (students & staff) - Introduction to the framework in Year 1. Revisit using experiential learning in “Forum Theatre” in Year 5 (38).	This provides an opportunity to reflect on unconscious biases and how to challenge and/advocate if experiencing/witnessing for example racism and sexism on campus and on clinical placement.
Online open-access EDI training, which is mandatory for all Family Medicine tutors (39).	Co-created between the two medical schools, and the postgraduate deanery in Northern Ireland, working alongside a medical student. This is deliberately freely available and open access for any interested parties globally (can be accessed through reference 39).
EDI training for clinicians, academics and Simulated Participants involved in medical student assessments.	Material addresses unconscious bias and stereotyping.
Half-day workshop on decolonising the curriculum attended by staff and students from Northern Ireland's two medical schools.	Aims of the session were to share good practice of decolonising the curriculum and to develop collaborations.
Learning practices which predated the C25 curriculum, and which align with a pedagogical cultural humility framework - Reflective Practice Portfolio as a continuum of self-reflection. - Interprofessional Schwartz rounds. - Involvement of real patients in Finals OSCEs (40). - Funded summer studentships for students to work alongside staff on EDI topics in relation to both education and research.	

The original version of this article has been updated.

